# Land use intensity has an impact on *Borrelia burgdorferi* sensu lato prevalence and genodiversity in ticks from Central Germany

**DOI:** 10.1186/s13071-025-06980-z

**Published:** 2025-09-19

**Authors:** Suscha Nicolina Lassen, Christian Imholt, Max Müller, Nina Król, Leonard Gothe, Lara Maria Inge Heyse, Martin Pfeffer, Anna Obiegala

**Affiliations:** 1 Institute of Animal Hygiene and Veterinary Public Health, Faculty of Veterinary Medicine, University of Leipzig, An Den Tierkliniken 1, 04103 Leipzig, Germany; 2 Institute for Epidemiology and Pathogen Diagnostics, Julius Kühn-Institute, Toppheideweg 88, 48161 Münster, Germany; 3 Institute of Evolutionary Ecology and Conservation Genomics, University of Ulm, Albert-Einstein-Allee 11, 89081 Ulm, Germany; 4 Fachgebiet 3.6 Virologie, Landesuntersuchungsanstalt für das Gesundheits- und Veterinärwesen Sachsen, Bahnhofstraße 58/60, 04158 Leipzig, Germany; 5 Institute of Ecology and Environmental Science of Paris, Sorbonne University, Campus Pierre et Marie Curie 44-45, 75005 Paris, France

**Keywords:** Ixodid ticks, *Borrelia*, Biodiversity, Grassland, Woodland, *Ixodes ricinus*, Habitat, Multilocus sequence typing, Anthropic pressure

## Abstract

**Background:**

Ticks are important vectors of zoonotic pathogens, with *Ixodes ricinus* being the most abundant and main vector in Europe of *Borrelia burgdorferi* sensu lato (s.l.), the causative agent of Lyme borreliosis. Both vector and reservoir hosts are dependent on habitat structure, which is anthropogenically influenced by land use intensity. This study aimed to analyse the prevalence of *B. burgdorferi* s.l. and their genodiversity in ticks along a land use gradient in grassland and forest in Central Germany.

**Methods:**

Ticks were collected from 25 grassland and 25 forest sites by using the flagging method and tested for *Borrelia * spp. using real-time polymerase chain reaction. Positive samples were further analysed by using multi-locus sequence typing to identify the exact *B. burgdorferi* s.l. genospecies and sequence types. To analyse the prevalence of *Borrelia* and the density of *I. ricinus*, confidence intervals, generalized linear mixed models, linear models, generalized linear models (Tweedie distribution), model selection (delta Akaike information criterion corrected for small sample size < 2), relative abundance index and the Shannon index were used.

**Results:**

In total, 210 of the 1896 ticks collected tested positive for *Borrelia* (11.08%). The prevalence in *I. ricinus* ticks was identical in females (48/156; 30.77%) and males (44/143; 30.77%) and lower in nymphs (118/1152; 10.24%). *Ixodes ricinus* collected from grassland were significantly more frequently infected (29.36%) than those from woodland (6.43%). A positive correlation between land use intensity and the infection rate of ticks with *B. burgdorferi* s.l. was found in both grassland and woodland. Furthermore, the relative abundance index of predatory and small mammals had a positive effect on *Borrelia* spp. prevalence in *I. ricinus* nymphs. Multilocus sequence typing was performed for 184 samples. The most frequently found genospecies was *Borrelia afzelii* (65.76%), followed by *Borrelia garinii* (17.93%), *Borrelia valaisiana* (13.59%), and *Borrelia burgdorferi* sensu stricto (2.72%). Furthermore, 59 known and 41 new sequence types were detected.

**Conclusions:**

*Borrelia burgdorferi* s.l. genotypes with zoonotic potential show variable host adaptation, which seems to promote high intraspecific pathogen diversity. The results of our study support the dilution hypothesis as they show that conserving native forests and species diversity may support the biodiversity of *Borrelia* spp. while reducing their overall prevalence.

**Graphical abstract:**

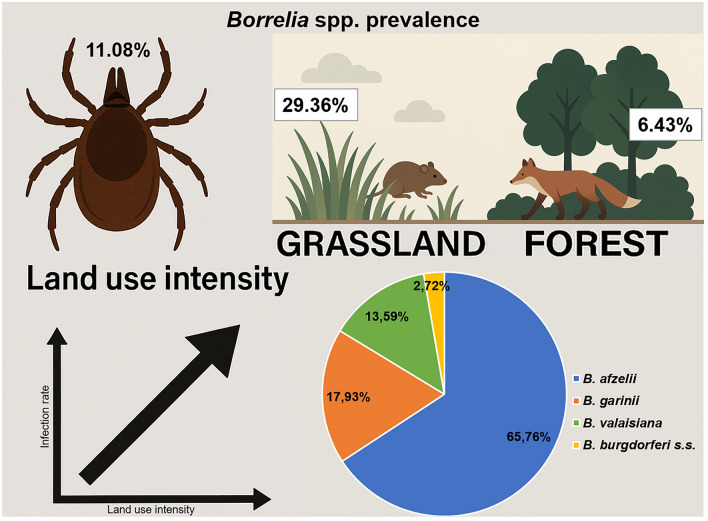

**Supplementary Information:**

The online version contains supplementary material available at 10.1186/s13071-025-06980-z.

## Background

Ticks are obligate bloodsucking parasites with the ability to transmit a variety of pathogens to both animals and humans. Hard ticks belong to the order Ixodida. Ixodida comprises three families, of which Ixodidae is the largest by number of species [1, 2]. The genus *Ixodes* comprises about 250 species, with the castor bean tick, *Ixodes ricinus,* being the most abundant one in Europe [3]. More than 300 different vertebrate species, from small rodents to large mammals, serve as hosts for *I. ricinus* [4]. Due to its broad host range and environmental plasticity, *I. ricinus* is considered a particularly important vector of various zoonotic pathogens, such as bacteria and viruses [5]. The most common tick-borne disease transmitted by *I. ricinus* in the Northern Hemisphere is Lyme borreliosis, an infectious multisystemic disease characterized by various symptoms that can be hard to treat and that may have a lethal outcome [6]. Lyme borreliosis is caused by species of the *Borrelia burgdorferi* sensu lato (s.l.) complex within the family Spirochaetaceae, which comprises Gram-negative, spiral-shaped, motile bacteria [7, 8]. Among the more than 20 identified genospecies of *B. burgdorferi* s.l., at least 11 are known to circulate in Europe [9]. Among these, the zoonotic *Borrelia afzelii* and *Borrelia garinii* are the most widespread [10].

Tick occurrence is influenced by the abundance and diversity of their hosts [11]. Host species may play different roles in a tick’s life cycle, and the reservoir competence of hosts of *Borrelia* genospecies varies [12, 13]. Animals such as white-footed mice, shrews, medium-sized mammals, and birds are competent *B. burgdorferi* s.l. reservoirs, while larger mammals such as deer, cattle, and raccoons are typically incompetent reservoirs [14–17]. The specific adaptation of different *Borrelia* species to certain vertebrate hosts has a major influence on their distribution and ecological function in complex host communities. This makes it difficult to reliably predict the frequency of infection [18]. The geographical spread and genetic diversity of *B. burgdorferi* s.l. are mainly determined by the presence of suitable reservoir hosts and the occurrence of certain tick species as competent vectors. Both of these factors are strongly dependent on habitat, which in turn is significantly influenced by external factors such as human land use and associated changes, e.g. in forest structure [19, 20]. Tick-borne pathogens therefore provide a meaningful model for analysing the effects of land use change on human and animal health, as they are directly influenced by anthropogenic activities [21].

To the best of our knowledge, this is the first study to examine *Borrelia* spp. and their genetic diversity in relation to the silvicultural management index (SMI), an established parameter used to assess forest use intensity. Exploring *Borrelia* genospecies diversity in Central Germany with regard to the intricacies of the ecology of this pathogen complex, their reservoir hosts and tick vectors, and the impact of anthropogenic activities on their diversity, should provide a better understanding of their life cycle and disease risk. As a similar study on *Borrelia* sequence types (STs) in ticks and small mammals conducted in the same region [18] showed high genodiversity in *Borrelia*, the current study focuses on the effects of land use on their genetic diversity.

The aims of the present study were, thus, to determine (1) the tick density and species composition of mammals, (2) the prevalence of *B. burgdorferi* s.l., and (3) the abundance of *Borrelia* genospecies and their genetic diversity in ticks along a predefined land use gradient in the Hainich-Dün region of northwestern Thuringia, Central Germany.

## Methods

### Collection sites

**Fig. 1:**
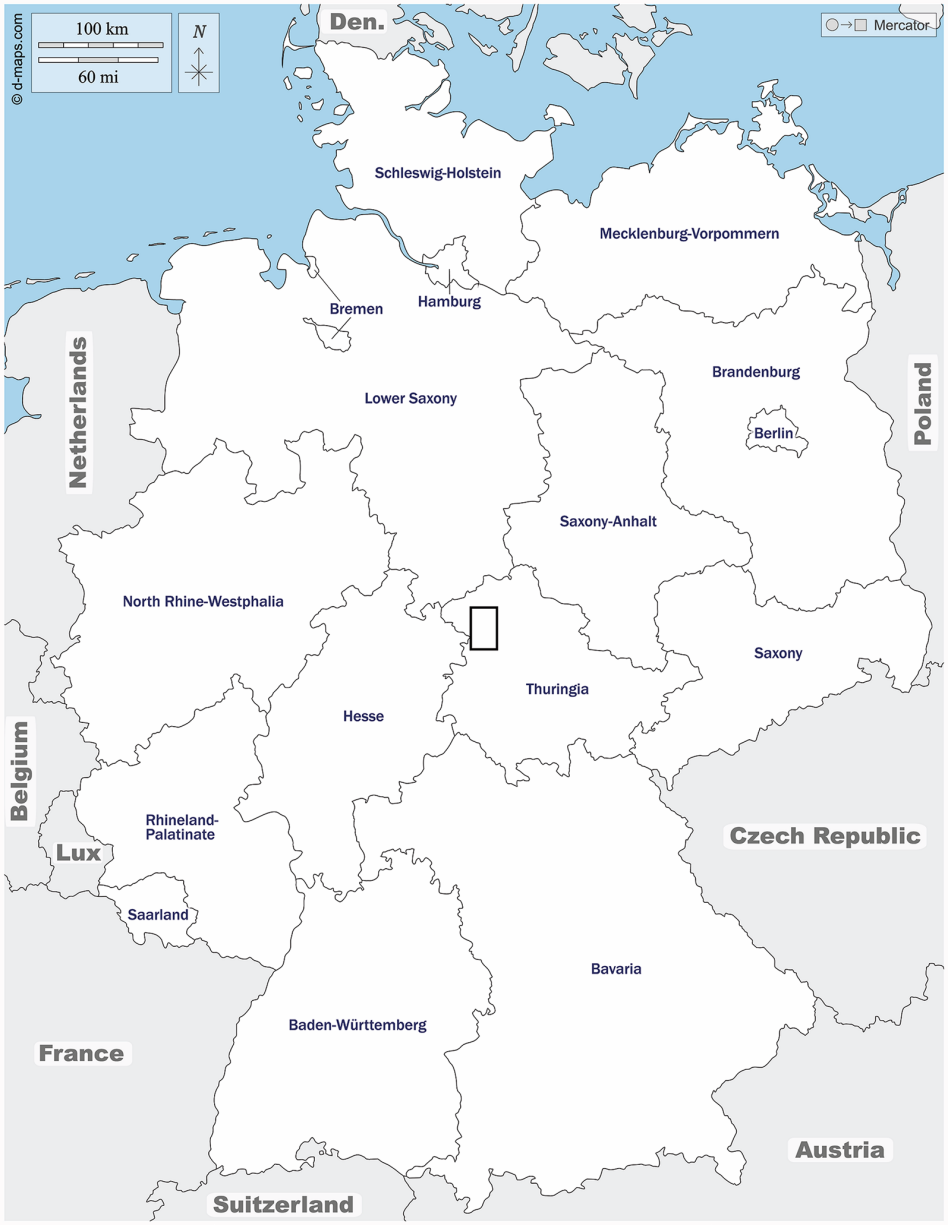
Map indicating the studied area (black rectangle) in Germany

The Biodiversity Exploratories research platform is an interdisciplinary project funded by the German Research Foundation (Deutsche Forschungsgemeinschaft; DFG). It consists of three long-term research areas—Schorfheide-Chorin, Hainich-Dün and the Swabian Alb—situated along a north–south axis that were established from 2006 to 2009 within a funded biodiversity research program. Each of these regions contains the majority of land use forms typical of grassland and forests in Germany and thus offer the opportunity to conduct comparative research on how the intensity of various types of land use affects biodiversity and ecosystem functions in actual managed areas [22]. For this study, the Hainich-Dün region (Fig. 1) [23], located in northwest Thuringia, Central Germany, was chosen as the research area. It covers approximately 1300 km^2^ and extends from the Hainich (51°6′N, 10°23′E), a forested chain of hills in the south, to the Dün (51°22′N, 10°15′E), a range of hills in the north. Hainich covers an area of about 160 km^2^, making it one of the largest areas of continuous deciduous forest in Germany. The Dün comprises a range of hills up to 522 m in elevation that cover an area of around 270 km^2^, about half of which is forested. In 1997, a 75-km^2^ area of Hainich was designated a national park. The vegetation of the mixed forest of Hainich is mainly characterized by deciduous forests, with European beech (*Fagus sylvatica*) comprising the majority of the trees and frequently present together with ash, sycamore maple, and elm, among others [19, 22]. In addition, agricultural land and extensively managed grasslands are also present.

**Fig. 2:**
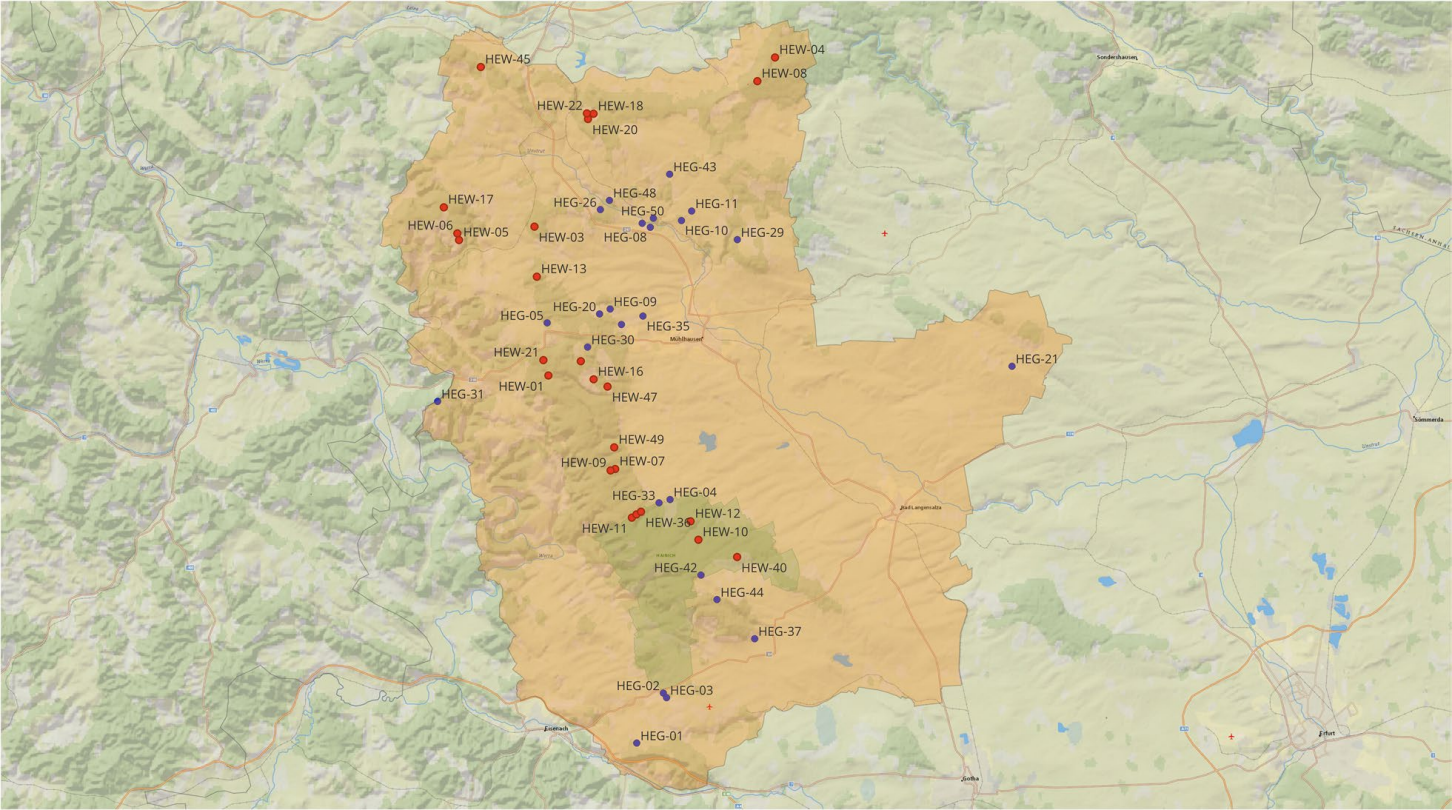
Locations of the tick collection sites (plots) in forests (red) and grasslands (blue) within the Hainich-Dün Biodiversity Exploratory (orange) (the map was drawn by using QGIS and includes the authors’ modifications).* HEW* Woodland,* HEG* Grassland

For the comparative study, 1000 grid plots (500 forest and 500 grassland plots) were designated that differed in terms of their management type. Of these, 100 sites of all land use and management types were randomly selected to conduct detailed biodiversity analysis and environmental monitoring. Fifty plots (25 sites in forest and 25 on grassland) covering the entire gradient of land use intensity in forest and grassland, and allowing for more labour-intensive research, were selected for our study (Fig. 2). The intensities of land/forest use ranges from unmanaged forest and sparsely used grassland to intensively used forests and grasslands [24].

A standardized measure of land use, a land use index (LUI), was determined for each grassland plot. The LUIs were calculated as grassland management indicators in accordance with Blüthgen et al. [25] by using the LUI calculation tool [26]. The LUI summarises the complexity of agricultural practices in a single quantitative indicator, providing a standardised basis for assessing how different groups of organisms and ecological processes respond to land use gradients and enabling the relative influence of anthropogenic land use to be assessed in comparison with other local or regional environmental factors. The LUI used here quantifies and reflects the three main components of grassland use: grazing, mowing and fertilization [27]. In addition, the SMI, a previously developed indicator for the intensity of forest management, was applied for each forest plot as a purely quantitative measure for classifying a set of forest stands along a management intensity gradient. This index is calculated from the risk component, which rates how likely it is that a forest stand will be lost before reaching a certain age, and the density component, which measures the deviation of stand density from that of a natural maximum. In the Hainich-Dün region, three main indicators were combined for the calculation of SMI: tree species, stand age, and living and dead woody biomass above ground [28].

### Questing tick collection and DNA extraction

Ticks were collected from vegetation once each season (except for winter), i.e. in June, September and November 2020 and in April, July and October 2021, by using the flagging method. Using this method, 150 m^2^ were flagged with a cotton flag in each plot (Fig. 2). The collected ticks were immediately preserved with 70% ethanol. To determine ticks to the species level, morphological identification [29, 30] was conducted under a stereomicroscope (SMZ171; MoticEurope, Barcelona, Spain). The ticks were rinsed twice in distilled water then once in phosphate-buffered saline (pH 7.4) and air dried before being stored individually at − 20 °C.

For the extraction of DNA, the ticks were first placed in homogenization tubes containing 1 g sterile steel beads (2.8-mm diameter; Peqlab Biotechnology, Erlangen, Germany) and 250 µl phosphate-buffered saline. The samples were then homogenised 3 times at 5500 r.p.m. for 15 s in a Precellys^®^24 tissue-homogenizer (Bertin Technologies, Montigny Le Bretonneux, France) with an interval of 10 s between each homogenisation.

The QIAamp^©^ DNA Mini Kit (QIAGEN, Hilden, Germany) was used as recommended by the manufacturer for the subsequent extraction of DNA. The quantity and quality of DNA were determined with a NanoDrop^®^ 2000c spectrophotometer (Peqlab Biotechnology). The samples were stored at − 80 °C until further experiments were carried out.

### Real-time polymerase chain reaction detection and multilocus sequence typing of* Borrelia burgdorferi* s.l.

For the detection of *Borrelia* spp. DNA, tick samples were initially analysed by real-time (quantitative) polymerase chain reaction (qPCR) targeting the *flagellin* gene (96 base pairs) based on a previously developed protocol using the QuantiTect Multiplex PCR NoROX kit (1000, 204745) (QIAGEN) [31, 32]. These reactions were performed using an Mx3000 Real-Time Cycler (Agilent, Santa Clara, CA). To identify the genospecies and genotypes of *B. burgdorferi* s.l., multilocus sequence typing (MLST) was performed, with specific primers adjusted for each of eight housekeeping genes, *recG, pyrG, uvrA, clpX, pepX, rplB, cplA,* and *nifS*, using the GoTaq^®^ G2 Hot Start Green Master Mix (Promega, Walldorf, Germany) with slight modifications (see Table S1) [33]. The MLST was performed for 187 samples with a cycle threshold value ≤ 37. PCR products were visualised by using the UVP GelSolo Simplified UV Gel Documentation System (Analytik Jena, Jena, Germany).

### PCR clean-up and sequence analysis

Positive PCR products were purified with the NucleoSpin^®^ Gel and PCR Clean-up Mini Kit (Macharey-Nagel, Düren, Germany) in accordance with the manufacturer’s recommendations. The purified products together with the primers matching each gene were sent to Eurofins Genomics (Ebersberg, Germany) for Sanger sequencing. Bionumerics (version 7.6.1; Applied Maths, Austin, TX) was used to analyse the results. The sequences of the eight housekeeping loci were compared with those published in the online MLST database (https://pubmlst.org/organisms/Borrelia-spp) to assign allele and ST profiles. Forty-one new allelic combinations were deposited in the PubMLST database under the following ST numbers: 1079, 1080 and 1082–1120.

For samples with incomplete allelic profiles, only the *Borrelia* genospecies were determined based on the *recG* gene using BLAST (https://blast.ncbi.nlm.nih.gov/Blast.cgi). We selected the *recG* gene for genotyping as it is present as a chromosomal gene in all of the strains and can be used to distinguish between *Borrelia* genospecies. Furthermore, it was the most reliable gene for genotyping.

### Mammal monitoring

Data from animal camera trapping and small mammal snap trapping were made available from a previous study undertaken by a member of our group [34]. Two camera traps per plot (Moultrie MCG-12181 M40 Game Camera and Moultrie MCG13271 M50 Game Camera; Schery, Fulda, Germany) were active for 2 months in each tick collection season (spring, summer and autumn in 2020 and 2021). The data were analysed and the species visually identified using Agouti.eu, a platform for the processing and archiving of camera trap images [35].

Rodent snap-trapping took place during the same time frame on two trap nights per season using 49 traps (DeuFa, Neuburg, Germany) in a 7 × 7-m grid per plot. For the detailed methodology, see Harpering [34].

### Statistical analysis

The 95% confidence intervals (CIs) for the *Borrelia* spp. prevalences were determined by the Clopper and Pearson method by using GraphPad software (San Diego, CA). To analyse *B. burgdorferi* s.l. prevalence in *I. ricinus* in relation to season, habitat, and LUI, we used a generalized linear mixed model (GLMM) with a binomial error distribution in R software (version 4.1.2. for Windows; RStudio, Boston, MA) and the lme4 package with a log logit function [36]. The GLMM was created to estimate if (1) seasonality (independent variable—spring, summer and autumn), (2) the independent variables *I. ricinus* development stage (binary—adult vs. immature), (3) habitat (independent binary variable—woodland vs. grassland), and (4) *I. ricinus* density had an influence on the individual infection status (dependent binary variable—*Borrelia* prevalence). Study site was used as a random factor.

Relative abundance indices (RAIs) were calculated in R 4.3.1 (16 June 2023) for each mammal species as events/trappings per 100 camera days/trap nights[37] with the script described in Rovero and Zimmermann [38].

$$RAI=\left(\frac{Number  \,of  \,detections \left(or\, captures \right) for\, a \,species}{Sampling \,effort \left(camera \,days \,or \,trap \,nights\right)}\right)\times 100$$

Data were averaged for each of the seasons. The main function of predators considered here is their control of the small mammal population. Large mammals maintain the tick population but are considered incompetent hosts for *B. burgdorferi* s.l. Larger (monitored with snap traps) and smaller non-predatory mammals (monitored with camera traps) are considered competent hosts for the pathogens and to maintain the tick population. Hence, for each animal group (predators, larger and smaller non-predatory mammals), the RAIs of the individual species were summarized and the Shannon index (*H*) calculated from the RAIs of each group ($H= \sum_{i}pi x\text{ln}pi$ where $pi= \frac{ni}{N}$,* N* = total sum of RAIs,* n*_*i*_ = sum of RAIs of respective species group; see Table S2).

Based on datasets elaborated from the Biodiversity Exploratories project, we tested the effects of local forest structure (cover of the herb and shrub layer [39]), climate variables (air temperature at 10-cm height and relative air humidity at 200-cm height [40]) and the mammal community (RAIs and Shannon diversity of small mammals, predators and smaller and larger non-predatory mammals [34, 41]) on *I. ricinus* density and *Borrelia* prevalence in *I. ricinus* (see Table S2).

For *I. ricinus* density as the response variable we fitted linear models. For *Borrelia* prevalence as the response variable we fitted generalized linear models with a Tweedie distribution. We conducted all analysis in R 4.3.1 (2023-06-16) [37]. Linear models were run using the lm function. Generalized linear models were run using the glmmTMB function of the glmmTMB package [42]. We selected the best-fitting models [delta Akaike information criterion corrected for small sample size (ΔAICc) < 2] and calculated their averages using the MuMIn package [43]. We report conditional averaged model results. All of the included candidate models are shown in the supplementary material (Tables S3, S4).

The values obtained for the autumn were not representative due to an unequal distribution of tick densities (which were lower in autumn) and were therefore not included in the GLMM. The number of ticks per season ranged from 210 to 940 ticks, which was considered a representative number of individuals for the statistical analyses.

## Results

### Tick collection

A total of 1896 ticks were collected. Three species were identified, with *Ixodes ricinus* being the most abundant (*n* = 1759; 92.77%), followed by *Dermacentor reticulatus* (*n* = 135; 7.12%) and *Ixodes frontalis* (*n* = 2; 0.11%). The most abundant life stage of *I. ricinus* found was the nymph stage (*n* = 1152; 65.49%), followed by the larval stage (*n* = 308; 17.51%); there were more females (*n* = 156; 8.87%) than males (*n* = 143; 8.13%) (Table 1).

**Table 1:** Number of ticks collected in the Hainich-Dün region

Tick species	Larvae	Nymphs	Females	Males	Total
*Ixodes ricinus*	308	1152	156	143	1759
*Dermacentor reticulatus*	0	0	72	63	135
*Ixodes frontalis*	0	2	0	0	2
Total	308	1154	228	206	1896

More than three times as many *I. ricinus* ticks were collected by flagging in the forest (*n* = 1341; 76.24%) compared to grassland (*n* = 418; 23.76%). Almost all of the *D. reticulatus* were found in grassland (132/135, 97.78%). Nearly half of the tick individuals were collected in spring (*n* = 940, 49.58%; of which *I. ricinus* *n* = 925, 98.4%; *D. reticulatus* *n* = 13, 1.38%; *I. frontalis* *n* = 2, 0.21%), followed by summer (*n* = 746; 39.35%; of which *I. ricinus* *n* = 30, 97.86%; *D. reticulatus* *n* = 16, 2.14%) and autumn (*n* = 210; 11.08%; of which *D. reticulatus* *n* = 106, 50.48%; *I. ricinus* *n* = 104, 49.52%). The tick density was 5.87 ticks per 150 m^2^ for *I. ricinus*, 0.45 ticks per 150 m^2^ for *D. reticulatus* and 0.007 ticks per 150 m^2^ for *I. frontalis*. The tick densities of the different life stages were as follows: 1.03 larvae per 150 m^2^, 3.85 nymphs per 150 m^2^ and 1.45 adults per 150 m^2^.

### *Borrelia *spp. prevalence in ticks

All of the collected ticks (*n* = 1896) were screened for *Borrelia* DNA. This was detected in 11.08% (95% CI 9.7–12.6) (*n* = 210) of the ticks via qPCR. All of the positive samples were from *I. ricinus* ticks (11.94%; 95% CI 10.5–13.6). The majority of *Borrelia*-positive ticks were nymphs (*n* = 118, 56.19%, 95% CI 49.2–63), followed by females (*n* = 48, 22.86%, 95% CI 17.4–29.1) and then males (*n* = 44) (20.95%, 95% CI 15.7–27.1). All larvae tested negative. The prevalence of *Borrelia* in adults (92/299, 30.77%, 95% CI 25.6–36.3) was equal between females (48/156, 30.77%, 95% CI 23.6–38.7) and males (44/143, 30.77%, 95% CI 23.3–39) (*p* = 1), and significantly higher than in nymphs (118/1152, 10.24%, 95% CI 8.6–12.1) (*p* = 0.0001). The GLMM showed that these differences were especially prominent in the spring (*p* = 0.000119) and less prominent in summer (*p* = 0.0112) (Table 2). The GLMM also showed that habitat had a highly significant positive influence on *Borrelia* prevalence in the spring (*p* = 0.011993), while in summer there was no influence of habitat (Table 2). The GLMM confirmed that nymphs in both grassland (*p* = 0.00974) and woodland (*p* = 0.00116) were significantly less often positive for *Borrelia* than adult ticks (Table 3; Table S5). There were no statistical differences in prevalence in collected ticks between 2020 (89/727, 12.24%, 95% CI 9.6—14.9) and 2021 (121/1169, 10.35%, 95% CI 8.7—12.2), (*p* = 0.2019). More ticks collected in the spring were infected with *Borrelia* (2020, 14.1%, 95% CI 11.3–17.3, 2021, 19.7%, 95% CI 15.9–23.9) than in the summer (2020, 7.97%, 95% CI 4.1–13.8). *Ixodes ricinus* ticks collected in grassland were significantly more often infected with *Borrelia* (123/418, 29.43%, 95% CI 25.1–34.1) than those from the forest plots (87/1341, 6.49%, 95% CI 5.2–7.9), (*p* = 0.00925) (Table 3; Table S5).

**Table 2:** Results of the generalized linear mixed model with a binomial error distribution with effects of season (excluding autumn), habitat and life stage of ticks (excluding larvae) on the probability of infection of *Ixodes ricinus* ticks with *Borrelia *

Factor	Estimate	SE	*z*-value	*p*-value
Spring				
Intercept	− 0.6909	0.4403	− 1.569	0.116594
LUI	0.1892	0.1896	0.998	0.318323
Stage female–male	− 0.1305	0.3025	− 0.431	0.666225
Stage female–nymph	− 0.9738	0.2531	− 3.848	0.000119***
Habitat grassland–wood	− 1.0337	0.4114	− 2.512	0.011993*
Summer				
Intercept	1.4665	1.6681	0.879	0.3793
LUI	− 0.8742	0.7341	− 1.191	0.2337
Stage female–male	− 1.0538	0.9788	− 1.077	0.2817
Stage female—nymph	− 1.9646	0.7746	− 2.536	0.0112*
Habitat grassland–wood	− 2.2420	1.3735	− 1.632	0.1026

*LUI* Land use index

*** p < 0.001, * *p* < 0.05

**Table 3:** Results of a generalized linear mixed model with a binomial error distribution with effects of location, seasonality, life stage of ticks, and LUI/silvicultural management index (*SMI*) on the probability of infection of *Ixodes ricinus* ticks (adults and nymphs) with *Borrelia*

Factor	Estimate	SE	*z*-value	*p*-value
Woodland without larvae				
Intercept	− 1.6292	0.5019	− 3.246	0.00117**
SMI	2.1786	1.1543	1.887	0.05912
Stage female–male	0.2475	0.4697	0.527	0.59834
Stage female—nymph	− 1.1928	0.3671	− 3.249	0.00116**
Season spring	− 0.3692	0.4257	− 0.867	0.38585
Season summer	− 0.2370	0.4424	− 0.536	0.59215
Grassland without larvae				
Intercept	− 0.2778	0.6459	− 0.430	0.66715
LUI	0.1445	0.1680	0.860	0.38992
Stage female–male	− 0.2216	0.3346	− 0.662	0.50772
Stage female—nymph	− 0.7422	0.2871	− 2.585	0.00974**
Season spring	− 0.4052	0.5752	− 0.704	0.48118
Season summer	− 1.0951	0.7087	− 1.545	0.12229
Habitat as factor without larvae				
Intercept	− 0.39867	0.49885	− 0.799	0.42419
LUI	0.15418	0.18369	0.839	0.40127
Stage female–male	− 0.03903	0.27669	− 0.141	0.88782
Stage female—nymph	− 0.94738	0.22814	− 4.153	3.29 × 10^–05^***
Habitat grassland-wood	− 0.99036	0.38050	− 2.603	0.00925**
Season spring	− 0.29429	0.33622	− 0.875	0.38142
Season summer	− 0.48127	0.36500	− 1.319	0.18732

*** p < 0.001, ** *p* < 0.01

### *Borrelia burgdorferi* s.l. genospecies and MLST analyses

MLST analysis was performed for 187 of the 210 *Borrelia*-positive samples. As none of the housekeeping genes could be amplified in three of these samples, they were excluded from the ST analysis. The *Borrelia* genospecies were determined based on the *recG* sequence data for 184 samples, 112 from grassland and 72 from forest. The most frequently detected genospecies was *B. afzelii* (*n* = 122; 66.3%), followed by *B. garinii* (*n* = 32; 17.39%), *B. valaisiana* (*n* = 26; 14.13%) and *B. burgdorferi* sensu stricto (s.s.) (*n* = 4; 2.17%) (Table 4).

**Table 4:** Frequency of detected *Borrelia burgdorferi* s.l. species in relation to tick life stage and occurrence in woodland and grassland

*Borrelia burgdorferi* s.l. genospecies and tick life stage	Proportion of samples per habitat (%; 95% CI)		
	Grassland	Woodland	Total
*Borrelia afzelii*	91/112 (81.25; 72.8–88.0)	31/72 (43.06; 31.4–55.3)	122/184 (66.3; 59.0–73.1)
Female	24/112 (21.43; 14.2–30.2)	3/72 (4.17; 0.9–11.7)	27/184 (14.67; 9.9–20.6)
Male	20/112 (17.86; 11.3–26.2)	3/72 (4.17; 0.9–11.7)	23/184 (12.5; 8.1–18.2)
Nymph	47/112 (41.96; 32.7–51.7)	25/72 (34.72; 23.9–46.9)	72/184 (39.13; 32–46.6)
*Borrelia garinii*	11/112 (9.82; 5.0–16.9)	21/72 (29.17; 19.1–41.1)	32/184 (17.39; 12.2–23.7)
Female	5/112 (4.46; 1.5–10.1)	5/72 (6.94; 2.3–15.5)	10/184 (5.43; 2.6–9.8)
Male	2/112 (1.79; 0.2–6.3)	4/72 (5.56; 1.5–13.6)	6/184 (3.26; 1.2–7.0)
Nymph	4/112 (3.57; 1–8.9)	12/72 (16.67; 8.9–27.3)	16/184 (8.7; 5.1–13.7)
*Borrelia valaisiana*	9/112 (8.04; 3.7–14.7)	17/72 (23.61; 14.4–35.1)	26/184 (14.13; 9.4–20.0)
Female	5/112 (4.46; 1.5–10.1)	5/72 (6.94; 2.3–15.5)	10/184 (5.43; 2.6–9.8)
Male	3/112 (2.68; 0.6–7.6)	4/72 (5.56; 1.5–13.6)	7/184 (3.8; 1.5–7.7)
Nymph	1/112 (0.89; 0.02 –4.9)	8/72 (11.11; 4.9–20.7)	9/184 (4.89; 2.3–9.1)
*Borrelia burgdorferi*** s.s.**	1/112 (0.89; 0.02–4.9)	3/72 (4.17; 0.9–11.7)	4/184 (2.17; 0.6–5.5)
Male	1/112 (0.89; 0.02 –4.9)	1/72 (1.39; 0.04–7.5)	2/184 (1.09; 0.13–3.9)
Nymph	0	2/72 (2.78; 3.4–9.7)	2/184 (1.09; 0.13–3.9)

Complete sequence typing was performed for 181 of the 184 samples, as in three of the samples not all of the eight housekeeping genes could be amplified. In total, 100 different STs were detected. For 116 (64.08%) samples, 59 different, previously identified STs were found; for the remaining 65 (35.92%) samples, 41 new STs were recorded (Table S6). The most commonly found ST, ST 347, belonging to *B. afzelii*, was found in 15 ticks from 14 different sites located in grassland and forest. The second most commonly found ST was the newly recorded *B. afzelii* ST 1080, found in 13 ticks from eight different sites. *Borrelia afzelii* ST 467 was detected in eight ticks from eight sites. The newly discovered ST 1105 belonging to *B. afzelii* was noted in seven ticks from three grassland sites, and *B. afzelii* ST 779 was found in six ticks from three grassland sites. Similarly, ST 988 (*B. afzelii*), which was detected four times, was only found in ticks from grassland. However, ST 199 (*B. valaisiana*), recorded four times, was only found in ticks from woodland plots. All of the other STs detected were found in four or fewer ticks (Table S7).

In grassland, 69 different STs were identified, whereas 45 STs were detected in woodland. The diversity of STs within the genospecies varied according to the number of samples collected. Specifically, *B. afzelii* exhibited 57 different STs across 122 samples, *B. garinii* 25 STs in 32 samples, *B. valaisiana* 15 STs in 26 samples, and *B. burgdorferi* s.s. three STs in four samples (Table S6).

### Influence of climatic factors, local management indices, forest structure, mammal abundance and diversity on tick density and *Borrelia burgdorferi* s.l. prevalence and diversity

**Fig. 3:**
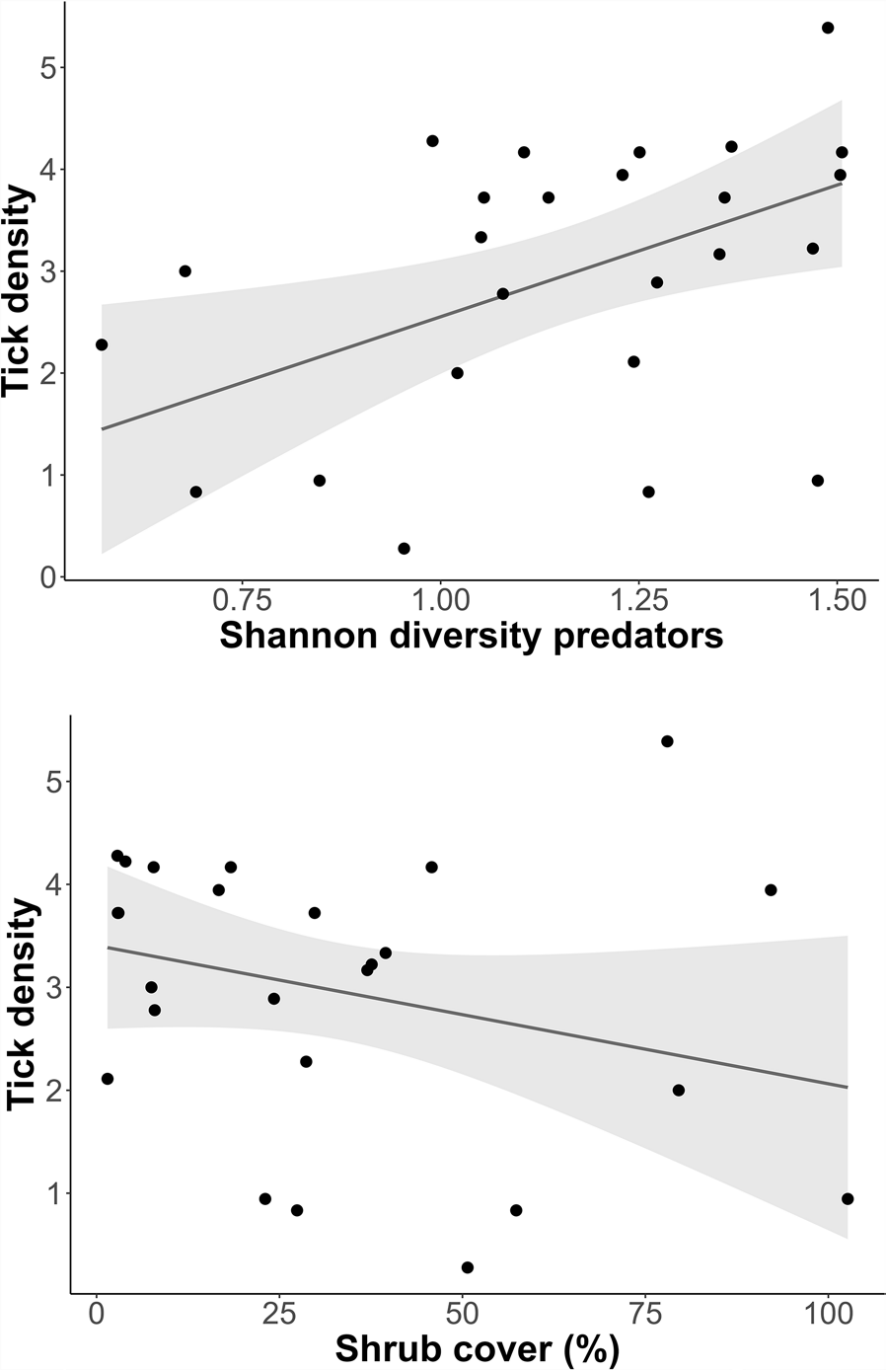
*Upper graph* The density of nymphs appears to be influenced by the diversity of the predator community.* Lower graph* The density of nymphs appears to be influenced by shrub cover as a trend

Model selection was performed using the dredge function followed by conditional averaging of the best-fitting models (ΔAICc < 2; Table S3). Of the 11 candidate predictors for tick density (see Table S2), only four (*H* predators, shrub cover,* H* small mammals, RAI larger non-predatory mammals; see Table 5) were retained in the conditional average model, of which only one was statistically significant (*H* predators), while another (shrub cover) showed a trend indicating that it had explanatory significance in predicting tick density. While the Shannon diversity index of predatory mammals had a significant positive influence on tick density (*p* = 0.0127), shrub cover had a negative influence on tick density (*p* = 0.0582) (Table 5; Fig. 3). Model selection followed by conditional averaging of the best-fitting models (ΔAICc < 2; Table S4) retained five candidate predictors (RAI predators, RAI small animals,* H* predators, shrub cover, air temperature) for *Borrelia* prevalence in the conditional average model (Table 6). However, the RAIs of predatory and small mammals had a positive influence on *Borrelia* prevalence (*p* = 0.0546, 0.0943) (Table 6), which suggests that the higher activity or frequency of these animal groups may contribute to the higher occurrence of the pathogen in the ecosystem.

**Table 5:** Statistical generalized linear model with four retained variables. The density of ticks appears to be influenced by the diversity of the predator community and – as a trend – by the shrub cover. (Full model: tick density ~ RAI small mammals + RAI predators + RAI smaller non-predatory mammals + RAI larger non-predatory mammals + H small mammals + H predators + H smaller & larger nonpredatory mammals + Herb cover + Shrub cover + air temperature + relative air humidity)

Factor	Estimate	SE	Adjusted SE	*z*-value	Pr( >|z|)
Tick density^a^					
Intercept	0.138044	1.503210	1.567249	0.088	0.9298
*H* predators	2.585699	0.984707	1.038138	2.491	0.0127 *
Shrub cover	− 0.017006	0.008474	0.008978	1.894	0.0582
*H* small mammals	1.533402	1.141831	1.211536	1.266	0.2056
RAI larger non-predatory mammals	− 0.001510	0.001340	0.001421	1.063	0.2879

*H* Shannon diversity index

* *p* < 0.05

^a^The density of ticks appeared to be influenced by the diversity of the predator community and—as a trend—by shrub cover. (Full model—tick density ~ relative abundance index (*RAI*) small mammals + RAI predators + RAI smaller non-predatory mammals + RAI larger non-predatory mammals + *H* small mammals + *H* predators + *H* smaller and larger non-predatory mammals + herb cover + shrub cover + air temperature + relative air humidity)

**Table 6:** Conditional averaging retained five variables in the model. *Borrelia* prevalence appeared to be positively influenced—as a trend—by the relative abundance of predatory mammals and of small mammals

Factor	Estimate	SE	Adjusted SE	*z*-value	Pr( >|*z*|)
*Borrelia* prevalence					
Intercept	0.448188	5.278201	5.405075	0.083	0.9339
RAI predators	0.004050	0.002002	0.002107	1.922	0.0546
RAI small mammals	0.008277	0.004641	0.004948	1.673	0.0943
*H* predators	− 1.287193	0.814272	0.866164	1.486	0.1373
Shrub cover	− 0.009212	0.006586	0.006988	1.318	0.1874
Ta_10	− 0.859046	0.505264	0.537631	1.598	0.1101

*Ta_10* Air temperature at 10 cm above ground level

Full model—*Borrelia* prevalence ~ RAI small mammals + RAI predators + RAI smaller non-predatory mammals + RAI larger non-predatory mammals + *H* small mammals + *H* predators + *H* smaller and larger non-predatory mammals + herb cover + shrub cover + air temperature + relative air humidity

**Fig. 4:**
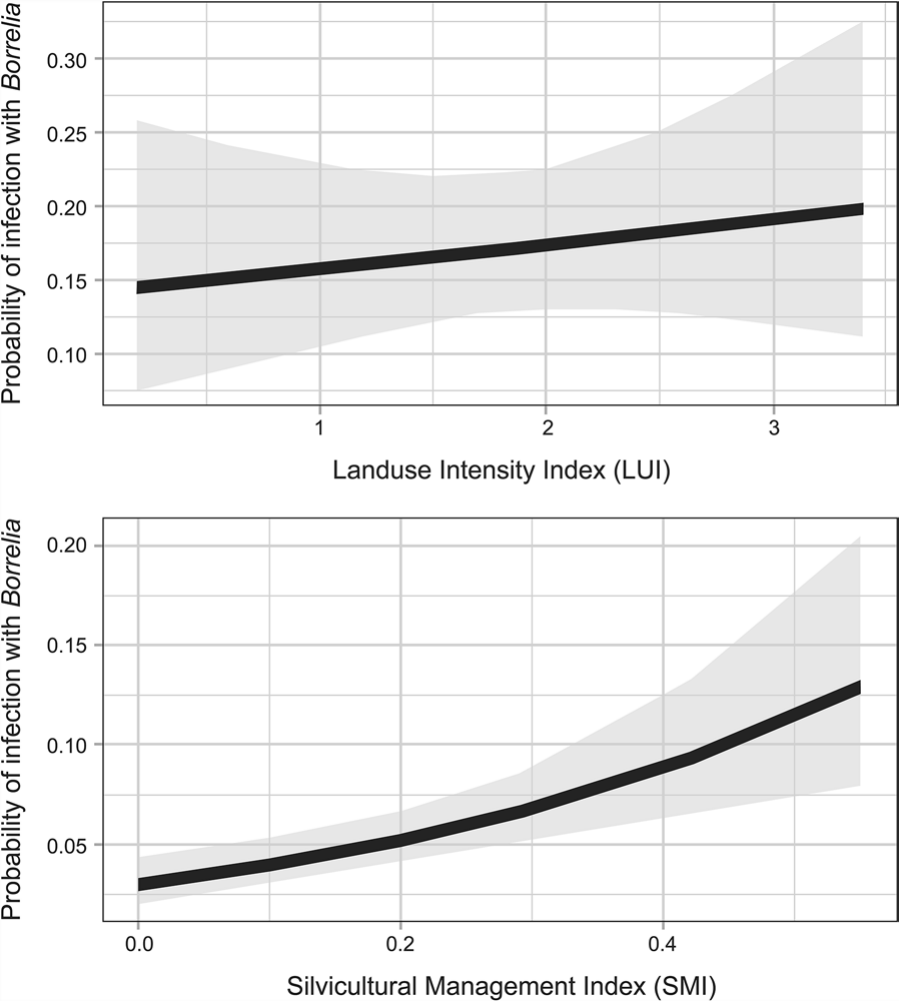
Probability of *Borrelia* infection plotted against land use index (LUI) (*upper graph*) and silvicultural management index (*lower graph*). Mean values fall within the area in grey. The relation between LUI and the probability of infection was not statistically significant. The relation between SMI and the probability of infection was slightly statistically significant

The SMI had no significant influence on tick density for the ticks found in forest. LUI for the grassland plots had no significant influence on *Borrelia* infection (*p* = 0.38992; Table 3; Fig. 4). The SMI had a slight influence (*p* = 0.05912) in the woodland plots on the infection of vectors with *Borrelia* (Table 3; Fig. 4). In summary, a higher LUI index was associated with increased tick density, while a higher SMI index was associated with higher *Borrelia* prevalence. The linear model revealed that *Borrelia* prevalence is positively linked to the number STs per site.

## Discussion

To the best of our knowledge, this is the first study to analyse *Borrelia* prevalence in *I. ricinus* not only along a land use gradient but also in relation to the SMI of forests. Three tick species were identified in this study, of which *I. ricinus* was the most common (92.77%), followed by *D. reticulatus* (7.12%) and *I. frontalis* (0.11%). These results are in line with those of previous studies undertaken in Europe [45, 46]. Similar to the present study, other work conducted in Central Europe [47], showed an increased activity of questing *I. ricinus* in the spring. That almost 50% of all of the ticks collected in the present study were found in the spring can be explained by local climatic factors such as temperature and humidity [48–50]. Two other studies undertaken in Europe also showed that the density and abundance of *I. ricinus* were related to climatic conditions [51, 52]. In the current study, no climatic factor were identified that influenced tick density. However, as the study design only allowed for limited spatiotemporal sampling, we were unable to extrapolate the data.

The abundance of *I. ricinus* was over three times higher in woodland than in grassland plots, which aligns with the findings of a meta-analysis undertaken using European data by Bourdin et al. [53]. That study reported greater ixodid tick abundance in forests, and attributed this to favourable conditions such as higher humidity and host species diversity [53]. Moist soil, a well-developed leaf layer, and the presence of beech trees further support tick density, particularly in the forests of Hainich-Dün [54, 55]. In the present study, only shrub cover was identified as a factor that negatively influenced tick density; however, it should be borne in mind that the flagging method is associated with errors, as thick shrub cover does not allow the same efficiency of flagging as clear ground does. The abundance of potential tick hosts likewise strongly influences tick density. For example, deer serve as a key host for all four tick life stages, and thus contribute to the proliferation of *I. ricinus* [56]. The model used in the current study revealed that the diversity of mammal predators, but not their relative abundance, has a significant effect on tick density. A possible reason for this might be that the predators, which can also be categorized as omnivores, serve as hosts for ticks but do not necessarily feed on small mammals due to their varied diet. A slightly statistically significant relationship was observed between the LUI of grassland plots and tick density; in contrast, no relationship between SMI and tick density was found for forests. Tick density is influenced by various factors, including survival rate, rates of development and reproduction, and dispersal. These are related in various ways to biotic and abiotic factors, some of which influence each other [5]. Intensive land use increases the abundance of key tick hosts, such as roe deer in forests and field voles in grasslands, which serve as tick multipliers. Thus intensified land use may facilitate the expansion of a tick population, as evidenced by the observed influence of land use in the present study.

The detected prevalence of *Borrelia* in *I. ricinus*, 11.94%, aligns with the that of studies undertaken in eastern, western, and southern Germany, which reported prevalences ranging from 9.4 to 34.1% [57–64]. Almost identical prevalence levels to those determined in the present study, i.e. 10.9% in 2022 [18] and 11.1% in 2003 [65], were reported for Thuringia and Hainich-Dün National Park. A meta-analysis [66] of *Borrelia* spp. prevalence in Europe reported an average infection rate of 13.6% across 155 studies. Unlike in studies undertaken in France, Poland, and other studies undertaken in Germany, the *D. reticulatus* collected and tested in our study were negative for *Borrelia*. This supports the theory that this tick species plays a subordinate role compared to *I. ricinus*, if any role at all, in the transmission of *Borrelia* [67–70].

Răileanu et al. [71] reported a higher prevalence of *B. burgdorferi* s.l. in forests, which was explained by their favourable conditions for tick vectors in combination with a high density of competent reservoir hosts that inhabited the forests. Interestingly, in the present study, the prevalence of *Borrelia* was higher in *I. ricinus* in the grassland plots than in the forest plots, despite a much lower tick density in grassland than in forest. These results indicate that the pathogen is transmitted more frequently in grassland, which was also reported in a previous study undertaken in 2022 in Hainich-Dün [18]. Studies on small vertebrates and pathogen prevalence conducted at the same sites within the Hainich-Dün Biodiversity Exploratory showed habitat preferences of small rodents, with the bank vole (*Clethrionomys glareolus*) and the yellow-necked mouse (*Apodemus flavicollis*) showing a preference for woodland and the common vole (*Microtus arvalis*) for grassland areas [72]. Studies have shown that *M. arvalis* is a competent reservoir host for *B. burgdorferi* s.l. [14, 72, 73], which explains the high prevalence of *B. burgdorferi* s.l. in the grassland areas of our study. The bank vole is a preferred host for ticks, as well as a suitable reservoir for *B. burgdorferi* s.l., and especially *B. afzelii*, and plays a role in the spread of these pathogens in beech-dominated forests [74, 75]. As also reported by Harpering [34, 41], we found that small mammals with a higher relative abundance—with the bank vole being by far the most common animal of this group in the present study—play a role in *Borrelia* prevalence [34, 41]. Larger vertebrates such as roe deer, which are considered incompetent hosts for *Borrelia* spp., are more likely to be found in thickets or forests than in open grassland. This may explain the significantly lower infection rate of *Borrelia* in ticks from woodland plots in our previous studies [18, 76], although differences in the abundances of larger mammals between the forest plots in the present study did not have a direct effect on the prevalence of *Borrelia*.

The absence of a measurable impact of LUI on *Borrelia* prevalence in grassland plots suggests that the management of these areas did not influence the infection rates in *I. ricinus* and is not the reason for the high prevalence, at 29.43%, of *Borrelia* in this habitat. The absence of a measurable impact of LUI on *Borrelia* prevalence may be explained by the low animal species richness reported in a previous study for grassland plots compared to forested ones [77]. One study [14] showed that the density of a host population influences how often pathogens and the host interact, thus the high prevalence of *M. arvalis* in the grassland plots of Hainich-Dün National Park in the present study may have led to an increase in the frequency of contact between the pathogen and this host, which in turn may partly explain the high incidence of *Borrelia* in the grassland habitat.

The significant correlation between SMI and *Borrelia* prevalence suggests that forest use and management influence rates of infection with *Borrelia.* The management of forests alters their vegetation structure, which in turn affects the complex interactions between ticks, hosts and pathogens. Numerous studies on land use and the risk of Lyme disease have focussed on the dilution effect hypothesis [78], according to which habitat disturbances reduce overall biodiversity, thereby promoting the proliferation of competent reservoir hosts and facilitating the transmission of *Borrelia* [20, 55, 76, 79, 80]. The positive correlation between SMI and the prevalence of *Borrelia* in vectors seen in the present study supports this model. This finding also supports the idea that preserving native forests and enhancing biodiversity may help to limit pathogen transmission [81]. However, in line with our results, in the latter study [81], it was not overall biodiversity but rather the RAI of small mammals and predators that was positively correlated with the prevalence of *Borrelia*. This suggests that local host density rather than species diversity in itself leads to higher infection rates—a trend that can again be interpreted as supporting the dilution effect hypothesis.

Four genospecies of *B. burgdorferi * s.l. were detected in the present study. *Borrelia afzelii* was by far thee most frequently detected species in *I. ricinus *ticks, followed by *B. garinii*, then *B. valaisiana,* and *B. burgdorferi* s.s. These results can be compared with those from previous studies undertaken in Europe, as all of these species have been detected there in the past, with *B. afzelii* and *B. garinii* most frequently found in ticks, followed by *B. valaisiana* and *B. burgdorferi* s.s. [10, 66, 82–84]. ST diversity was higher in grasslands than in forests and higher in adults compared to nymphs. This, along with the greater *B. burgdorferi* s.l. prevalence in grasslands, likely resulted from increased transmission by competent hosts driving ST variation within the genospecies. Adult ticks probably harbour a greater diversity of *Borrelia* STs because they feed on a wide variety of hosts multiple times. New STs arise through genetic heterogeneity, which is driven by mutations and recombination in housekeeping genes. The high ST diversity of *B. afzelii* is linked to its genetic variability and adaptation to reservoir hosts, where colonization fosters the emergence of new STs. Additionally, the geographical spread of the host or its vector(s) contributes to the emergence and establishment of new STs [33, 85–87].

Clinical manifestations of Lyme borreliosis in humans vary by genospecies; *B. afzelii* is commonly linked to skin manifestations, while neuroborreliosis is often associated with *B. garinii* [84, 88]. In the present study, genospecies prevalence differed by habitat, with *B. afzelii* more common in grasslands and *B. garinii* and *B. valaisiana* more frequent in woodlands. These distributions align with host adaptation, as previous studies showed an association between *B. garinii* and *B. valaisiana* and forest-dwelling songbirds, while *B. afzelii* is primarily transmitted by small rodents, which are abundant in Hainich-Dün National Park, and may explain its dominance in our study. Additionally, hedgehogs and Eurasian red squirrels serve as reservoirs for *B. afzelii* [18, 88–91].

In total, 59 previously identified STs were identified in the current study, of which 19 had already been found in Germany and Europe, both in ticks and in humans. Nine of the 59 STs have been isolated from ticks, humans, and animal hosts only in Germany, while 31 have been found in ticks and humans in Europe, but have never been recorded in Germany before [92]. In the present study, some pathogenic STs were detected that have been previously shown to cause symptoms in humans. ST 482 (*B. garinii*), which was detected once in our study, has been isolated from a patient with neuroborreliosis. The *B. afzelii* STs 774 and 779, which were detected in seven of our samples, has been previously isolated from patients with erythema migrans. Until now, ST 1034 belonging to *B. afzelii* has only been detected in humans, not in ticks or animal hosts [92]. One of the most often detected STs (*n* = 8) in our study, ST 467 (*B. afzelii*), has been previously found only in infected humans, and may be associated with erythema migrans. Also, two other *B. afzelii* STs, ST 1034 (detected once in our study) and ST 476 (found in four of our samples), have been previously found only in humans, in which they caused acrodermatitis chronica atrophicans [93].

The fact that 100 STs were found in 181 samples in the present study (including 41 newly discovered STs) shows that there is a high mutation rate in *Borrelia* in the Hainich-Dün region. Studies investigating the virulence of various STs have shown that there are not only inter- but also intraspecific differences in infectivity characteristics within *B. burgdorferi* s.l. [86]. The high number of STs detected in Hainich-Dün National Park, which have either known or unknown zoonotic potential, indicates the presence of a high number of hosts of varying potential as reservoirs [76, 86, 93, 94].

## Conclusions

The identification of 100 STs in the study sites indicates a high incidence and genotype diversity of *B. burgdorferi* s.l. in the Hainich-Dün region. This heterogeneity likely stems from a diversity of reservoir hosts and active recombination within the genospecies. Tick density was higher in forests than grasslands, yet *Borrelia* prevalence was greater in grasslands. This was likely due to the efficient transmission of the pathogen by hosts like *M. arvalis*, which is commonly found in the Hainich-Dün region. Forest use influences *Borrelia* prevalence in *I. ricinus* and impacts tick density by altering biodiversity and host availability. Given the zoonotic potential of the *B. burgdorferi* s.l. genospecies detected in the present study, the findings reported here should be helpful for further assessment of the impacts of different types of land use on ecosystems, biodiversity, and the risk of Lyme borreliosis in different habitats. The conservation of native forests and their species diversity should support biodiversity while reducing the prevalence of *Borrelia*.

## Data availability 

This work is based on data elaborated by several projects of the Biodiversity Exploratories program (DFG Priority Program 1374). The datasets are publicly available in the Biodiversity Exploratories Information System (10.17616/R32P9Q) and are listed in the references [39404144]. Also see appendix Table S2.

## Supplementary Information

Supplementary Material 1: Table S1. Amplification thermoprofile of MLST (first and second reactions of nested PCRs) for the eight housekeeping genes. Table S2. Number of ticks infected and *Borrelia burgdorferi* s.l. species found in the Hainich-Dün region divided by year, season and habitat. Table S3. Diversity of STs within each *Borrelia burgdorferi* s.l. genospecies. Table S4. List and frequency of five most common STs found in predominant genospecies, *B. afzelii* . Table S5. STs, *Borrelia* genospecies and their frequence of detection .

## Data Availability

This work is based on data elaborated by several projects of the Biodiversity Exploratories program (DFG Priority Program 1374). The datasets are publicly available in the Biodiversity Exploratories Information System (10.17616/R32P9Q) and are listed in the references [39, 40, 41, 44]. Also see appendix Table S2.

## Abbreviations

CI: Confidence interval
DFG: Deutsche Forschungsgemeinschaft (German Research Foundation)
GLMM: Generalized linear mixed model
LUI: Land use index
MLST: Multilocus sequence typing
PCR: Polymerase chain reaction
qPCR: Quantitative (real-time) polymerase chain reaction
RAI: Relative abundance index
s.l.: Sensu lato
SMI: Silvicultural management index
s.s.: Sensu stricto
ST: Sequence type
