# Supplementary material for: Land use intensity has an impact on Borrelia burgdorferi sensu lato prevalence and genodiversity in ticks from Central Germany
Source: Parasit Vectors. 2025 Sep 19;18:371. doi: 10.1186/s13071-025-06980-z (PMC12449800; doi:10.1186/s13071-025-06980-z)
Supplement: Supplementary file 1 — Supplementary Material 1: Table S1. Amplification thermoprofile of MLST (first and second reactions of nested PCRs) for the eight housekeeping genes. Table S2. Number of ticks infected and Borrelia burgdorferi s.l. species found in the Hainich-Dün region divided by year, season and habitat. Table S3. Diversity of STs within each Borrelia burgdorferi s.l. genospecies. Table S4. List and frequency of five most common STs found in predominant genospecies, B. afzelii . Table S5. STs, Borrelia genospecies and their frequence of detection . [file 13071_2025_6980_MOESM1_ESM.docx]

**Supplementary files S**

**Table S1** Amplification thermoprofile of MLST (first and second reactions of nested PCRs) for the eight housekeeping genes.

| **Gene** | **Temperature of annealing (°C) in** | |
| --- | --- | --- |
|  | **1. amplification** | **2. amplification** |
| *nifS* | 51°C-43°C  touchdown* | 55°C |
| *pyrG* | 47°C | 49°C |
| *clpX* | 56°C-46°C  touchdown** | 56°C-46°C  touchdown** |
| *pepX* | 56°C-46°C  touchdown** | 55°C |
| *uvrA* | 51°C | 51°C |
| *rplB* | 56°C-46°C  touchdown** | 56°C-46°C  touchdown** |
| *clpA* | 47°C | 56°C-46°C  touchdown** |
| *recG* | 55°C | 50°C |

* Temperature decrease of 1°C per cycle from 51°C to 43°C for first 9 cycles, 43°C afterwards

** Temperature decrease of 1°C per cycle from 56°C to 46°C for 9 cycles, 46°C afterwards

**Table S2** Number of ticks infected and *Borrelia burgdorferi* s.l. species found in the Hainich-Dün region

divided by year, season and habitat

| **Total** | **Year** | **Season** | **Habitat** | **No. of *B.* *burgdorferi* s.l. species  occurrence/total (%)** |
| --- | --- | --- | --- | --- |
| **No. of infected ticks/total**  **(%; 95% CI)** | | | |  |
| 210/1896  (11.08; 9.7 – 12.6) | 2020  89/727  (12.24; 9.6 – 14.9) | Spring  76/539  (14.10; 11.3 – 17.3) | Grassland  45/136  (33.09; 25.3 – 41.7) | *B. afzelii* 36/45 (80) |
|  |  |  |  | *B. garinii* 1/45 (0.02) |
|  |  |  |  | *B. valaisiana* 5/45 (11.1) |
|  |  |  |  | *B. burgdorferi* s.s 1/45 (0.02) |
|  |  |  | Woodland  31/403  (7.69; 5.3 – 10.7) | *B. afzelii* 9/31(29.03) |
|  |  |  |  | *B. garinii*  13/31 (41.94) |
|  |  |  |  | *B. valaisiana*  6/31 (19.35) |
|  |  |  |  | *B. burgdorferi* s.s*.*  1/31 (3.23) |
|  |  | Summer  11/138  (7.97; 4.1 – 13.8) | Grassland  2/29  (6.9; 0.9 – 22.8) | *B. afzelii* 1/29 (3.45) |
|  |  |  | Woodland  9/109  (8.26; 3.9 – 15.1) | *B. afzelii* 4/9 (44.44) |
|  |  |  |  | *B. garinii* 1/9 (11.11) |
|  |  |  |  | *B. valaisiana* 2/9 (22.22) |
|  |  | Fall  2/50  (4; 0,5 – 13.7) | Grassland  2/40 (5; 0.6 – 16.9) | *B. afzelii* 1/2 (50) |
|  |  |  |  | *B. garinii* 1/2 (50) |
|  |  |  | Woodland  0/10 | - |
|  | 2021  121/1169  (10.35; 8.7 – 12.2) | Spring  79/401  (19.7; 15.9 – 23.9) | Grassland  64/228  (28.07; 22.3 – 34.4) | *B. afzelii* 46/64 (71.88) |
|  |  |  |  | *B. garinii* 8/64 (12.5) |
|  |  |  |  | *B. valaisiana* 3/64 (4.69) |
|  |  |  | Woodland  15/173  (8.67; 4.9 – 13.9) | *B. afzelii* 4/15(26.67) |
|  |  |  |  | *B. garinii* 3/15 (20) |
|  |  |  |  | *B. valaisiana* 1/15 (6.67) |
|  |  | Summer  29/608  (4.77; 3.2 – 6.8) | Grassland  5/32  (15.63; 5.3 – 32.8) | *B. afzelii* 2/5 (40) |
|  |  |  |  | *B. garinii* 1/5 (20) |
|  |  |  |  | *B. valaisiana* 1/5 (20) |
|  |  |  | Woodland  24/576  (4.17; 2.7 – 6.1) | *B. afzelii* 12/24 (50) |
|  |  |  |  | *B. garinii* 3/24 (12.5) |
|  |  |  |  | *B. valaisiana*  5/24 (20.83) |
|  |  |  |  | *B. burgdorferi* s.s  1/24 (4.17) |
|  |  | Fall  13/160  (8.13; 4.4 – 13.5) | Grassland  5/85  (5.88; 1.9 – 13.2) | *B. afzelii* 5/5 (100%) |
|  |  |  | Woodland  8/75  (10.67; 4.7 – 19.9) | *B. afzelii* 2/8 (25) |
|  |  |  |  | *B. garinii* 1/8 (12.5) |
|  |  |  |  | *B. valaisiana* 3/8 (37.5) |
|  |  |  |  | *B. burgdorferi* s.s 1/8 (12.5) |

**Table S3** Diversity of STs within each *Borrelia burgdorferi* s.l. genospecies

| ***Borrelia* *burgdorferi* genospecies** | **Number of different sequence types detected** |
| --- | --- |
| *Borrelia afzelii* | 57 |
| *Borrelia garinii* | 25 |
| *Borrelia valaisiana* | 15 |
| *Borrelia burgdorferi* s.s. | 3 |

**Table S4** List and frequency of five most common STs found in predominant genospecies, *B. afzelii*

| **ST** | **No. of ticks infected** | **No. of different plots where ST was found** | | |
| --- | --- | --- | --- | --- |
|  |  | **Total** | **Woodland** | **Grassland** |
| 347 | 15 | 14 | 4 | 11 |
| 1080 | 13 | 8 | 5 | 8 |
| 467 | 8 | 8 | 6 | 2 |
| 1105 | 7 | 3 | 0 | 7 |
| 779 | 6 | 3 | 0 | 6 |

**Table S5** STs, *Borrelia* species and their frequence of detection

| **ST** | **Frequency of detection** | ***Borrelia burgdorferi s.l.* species** |
| --- | --- | --- |
| 20 | **2** | *B. burgdorferi* s.s. |
| 24 | **1** | *B. burgdorferi* s.s. |
| 27 | **1** | *B. burgdorferi* s.s. |
| 75 | **1** | *B. afzelii* |
| 82 | **1** | *B. garinii* |
| 86 | **2** | *B. garinii* |
| 94 | **2** | *B. garinii* |
| 96 | **2** | *B. valaisiana* |
| 97 | **4** | *B. valaisiana* |
| 98 | **1** | *B. valaisiana* |
| 102 | **2** | *B. valaisiana* |
| 168 | **1** | *B. afzelii* |
| 170 | **1** | *B. afzelii* |
| 171 | **2** | *B. afzelii* |
| 175 | **1** | *B. garinii* |
| 184 | **2** | *B. garinii* |
| 187 | **1** | *B. garinii* |
| 199 | **4** | *B. valaisiana* |
| 200 | **1** | *B. valaisiana* |
| 203 | **2** | *B. valaisiana* |
| 204 | **2** | *B. afzelii* |
| 206 | **1** | *B. valaisiana* |
| 207 | **1** | *B. garinii* |
| 211 | **1** | *B. valaisiana* |
| 212 | **3** | *B. valaisiana* |
| 243 | **1** | *B. garinii* |
| 244 | **1** | *B. garinii* |
| 245 | **2** | *B. garinii* |
| 246 | **1** | *B. garinii* |
| 251 | **2** | *B. garinii* |
| 258 | **3** | *B. afzelii* |
| 263 | **1** | *B. afzelii* |
| 289 | **1** | *B. afzelii* |
| 343 | **1** | *B. afzelii* |
| 347 | **15** | *B. afzelii* |
| 459 | **1** | *B. afzelii* |
| 467 | **8** | *B. afzelii* |
| 476 | **4** | *B. afzelii* |
| 482 | **1** | *B. garinii* |
| 484 | **1** | *B. afzelii* |
| 679 | **1** | *B. afzelii* |
| 705 | **2** | *B. afzelii* |
| 710 | **1** | *B. afzelii* |
| 743 | **2** | *B. garinii* |
| 753 | **1** | *B. afzelii* |
| 774 | **1** | *B. afzelii* |
| 779 | **6** | *B. afzelii* |
| 824 | **1** | *B. valaisiana* |
| 896 | **1** | *B. afzelii* |
| 936 | **1** | *B. garinii* |
| 937 | **1** | *B. afzelii* |
| 945 | **1** | *B. afzelii* |
| 987 | **1** | *B. afzelii* |
| 988 | **4** | *B. afzelii* |
| 993 | **1** | *B. afzelii* |
| 994 | **1** | *B. afzelii* |
| 997 | **1** | *B. afzelii* |
| 998 | **3** | *B. afzelii* |
| 1034 | **1** | *B. afzelii* |
| 1079 | **1** | *B. afzelii* |
| 1080 | **13** | *B. afzelii* |
| 1082 | **1** | *B. afzelii* |
| 1083 | **3** | *B. afzelii* |
| 1084 | **1** | *B. afzelii* |
| 1085 | **1** | *B. afzelii* |
| 1086 | **1** | *B. garinii* |
| 1087 | **1** | *B. valaisiana* |
| 1088 | **1** | *B. garinii* |
| 1089 | **1** | *B. garinii* |
| 1090 | **1** | *B. garinii* |
| 1091 | **1** | *B. garinii* |
| 1092 | **1** | *B. garinii* |
| 1093 | **1** | *B. valaisiana* |
| 1094 | **1** | *B. valaisiana* |
| 1095 | **1** | *B. valaisiana* |
| 1096 | **1** | *B. afzelii* |
| 1097 | **3** | *B. afzelii* |
| 1098 | **1** | *B. afzelii* |
| 1099 | **2** | *B. afzelii* |
| 1100 | **1** | *B. afzelii* |
| 1101 | **1** | *B. afzelii* |
| 1102 | **1** | *B. garinii* |
| 1103 | **1** | *B. garinii* |
| 1104 | **1** | *B. garinii* |
| 1105 | **7** | *B. afzelii* |
| 1106 | **1** | *B. afzelii* |
| 1107 | **1** | *B. afzelii* |
| 1108 | **1** | *B. afzelii* |
| 1109 | **1** | *B. afzelii* |
| 1110 | **1** | *B. afzelii* |
| 1111 | **2** | *B. afzelii* |
| 1112 | **1** | *B. afzelii* |
| 1113 | **1** | *B. afzelii* |
| 1114 | **1** | *B. afzelii* |
| 1115 | **1** | *B. garinii* |
| 1116 | **1** | *B. afzelii* |
| 1117 | **1** | *B. afzelii* |
| 1118 | **1** | *B. afzelii* |
| 1119 | **1** | *B. afzelii* |
| 1120 | **1** | *B. afzelii* |
